# αV Integrin Induces Multicellular Radioresistance in Human Nasopharyngeal Carcinoma via Activating SAPK/JNK Pathway

**DOI:** 10.1371/journal.pone.0038737

**Published:** 2012-06-13

**Authors:** Juanjuan Ou, Wei Luan, Jia Deng, Rina Sa, Houjie Liang

**Affiliations:** Department of Oncology and Southwest Cancer Center, Southwest Hospital, Third Military Medical University, Chongqing, People’s Republic of China; The Chinese University of Hong Kong, Hong Kong

## Abstract

**Background:**

Tumor cells acquire the capacity of resistance to chemotherapy or radiotherapy via cell–matrix and cell–cell crosstalk. Integrins are the most important cell adhesion molecules, in which αV integrin mainly mediating the tight contact between tumor cells.

**Methodology/Principal Findings:**

To investigate the role of αV integrin in multi-cellular radioresistance (MCR) of human nasopharyngeal carcinoma (NPC), we performed immunohistochemistry and Western blotting to find that the expression of αV integrin in the tumor tissue of radioresistant patients is much higher than that in radiosensitive patients. In vitro, we cultured human NPC cell line CNE-2 cells as multi-cellular spheroids (MCSs) or as monolayer cells (MCs), and found that the expression of αV integrin in MCSs is significantly higher than that in MCs. MTT, flow cytometry and clonogenic suvival assays showed that MCSs are less sensitive to X-ray irradiation than MCs while blocking of αV integrin in MCSs dramatically reversed their radioresistance. Furthermore, as detected by Western blotting, MCSs displayed sustained activation of the stress-activated protein kinase/c-Jun NH2-terminal kinase (SAPK/JNK) pathway in presence of irradiation. Blocking of αV integrin in MCSs decreased the expression of phosphorylated JNK. Additionally, blocking of SAPK/JNK signaling pathway synergistically induced apoptosis of MCSs exposed to irradiation by increasing the expression of cleaved caspase-3. In vivo, we found that irradiation combined with αV integrin blocking treatment significantly enhanced the radiosensitivity of NPC xenografts.

**Conclusions:**

Our results indicate a novel role of αV integrin in multi-cellular radioresistance of NPCs.

## Introduction

Nasopharyngeal carcinoma is the most common malignancy of head and neck in Southeast Asia, and radiotherapy is the most effective treatment [1]. Nevertheless, radioresistance still occurs in a high proportion of NPC patients, which is the main risk factor contributing to poor prognosis [2]. Thus understanding of the molecular mechanisms underlying radioresistance may provide opportunity to develop more effective anti-cancer strategy. The previous researches about tumor radiosensitivity mainly focus on a single tumor cell, disregarding the fact that in tumor mass, tumor cells acquire some new characteristics by interacting with each other to become more resistant to chemo- or radiotherapy, termed multi-cellular resistance (MCR) [3].

Integrins are critical cell adhesion molecules mediating the crosstalk between tumor cells and participating in cell invasion, metastasis, angiogenesis, cell survival and some other important biological behaviors of tumor cells [4]–[8]. More specifically, αV integrin is expressed in most cancer cells playing an essential role mediating cell–matrix and cell–cell interactions. Meanwhile, αV integrin is a key molecule contributing to cell proliferation and apoptosis [9]–[11]. Given the correlations between apoptosis and radiosensitivity, We then hypothesized that αV integrin may act as a pivotal factor inducing radioresisitance in NPCs.

In this study, we tested the hypothesis that αV integrin may cause multi-cellular radioresistance of NPC in a three-dimensional culture condition mimicking a tumor microenvironment, and we found that αV integrin expression is required for sustaining multi-cellular radioresistance in human NPC cell line CNE-2. Furthermore, we demonstrated that SAPK/JNK signaling pathway was involved in αV integrin mediated radioresistance. Our finding for the first time shows the important role of αV integrin in multi-cellular radioresistance of nasopharyngeal carcinomas.

## Results

### αV Integrin is Highly Expressed in NPC Tissues of Radioresistant Patient, and is Correlated to the Expressions of Apoptosis Related Genes

**Figure 1:**
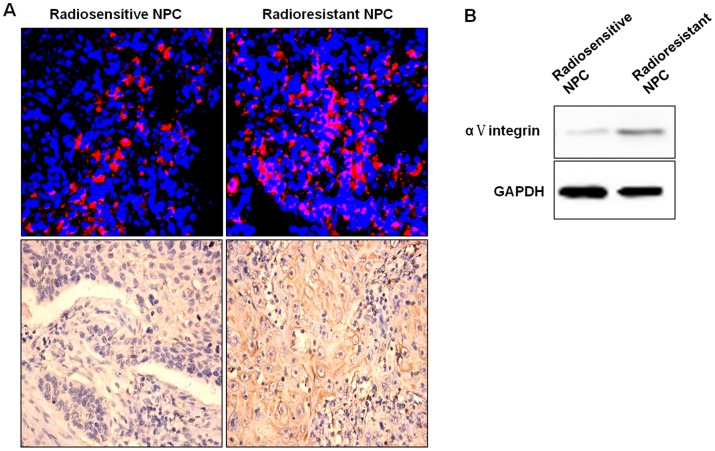
The expressions of αV integrin in NPC tumor tissues of radioresistant patients and radiosensitive patients. **A:** The immunoflurorescence and immunohistochemistry staining of αV integrin in the tumor tissues of NPC patients. (×100). **B:** The protein expression of αV integrin of NPC tumors.

To determine whether the expressions of αV integrin of NPC tumors are different in patients with different radiosensitivity, immunohistochemical technique was performed to detect the expressions of αv integrin in the 105 cases of tumor tissues and 20 cases of adjacent tissues. The positive expressions of αv integrin in NPC tumor tissues were shown to be significantly higher than those in the adjacent tissues. The expression of αv integrin are correlated to the differentiation degree of cancer cells and lymph node metastases (p<0.01), but not correlated to the patient’s gender, age, tumor location or tumor size (p>0.05) (Table 1). We also found that the expressions of αV integrin in radioresisitant patients are much higher than those of radiosensitive patients (Figure 1 A, B) and the levels of αv integrin are highly correlated with the Objective Response Rate (ORR) of NPCs (Table 2). Given apoptosis is an unarguably common pathway to cell death initiating from irradiation. We speculate that αV integrin may affect the levels of apoptotic genes. We therefore measured the expressions of cleaved Caspase-3 and cleaved PARP in these 105 cases of NPC patients, and found that the expressions of αV integrin are negatively correlated with the levels of cleaved Caspase-3 and PARP (P<0.01) (Table 3).

**Table 1:** The correlation between αV integrin expression and clinicopathological features in nasopharyngeal carcinomas.

Variables	No. patient	αV Integrin expression		*P* value
		−/±	+/++	
malignant tissue	105	47	58	0.004
Normal tissue	20	16	4	
**Age (years)**				0.344
≤50	41	16	25	
>50	64	31	33	
**Gender**				0.743
Male	71	31	40	
Female	34	16	18	
**Location**				0.889
Pharynx	23	10	13	
Larynx	82	37	45	
**Differentiation**				0.015
well+moderate	72	38	34	
poor+nondifferentiated	33	9	24	
**Nodal status**				0.027
Negative	59	32	27	
Positive	46	15	31	

**Table 2:** The correlation between αV integrin and objective response rate.

ORR	Number of patients		*P* value
	αV integrin low expression	αV integrin high expression	
CR	55	6	<0.001
PR+SD	13	31	
RLN	8	29	<0.01
NRLN	60	8	

**RRLN:** Residual of Cervical Lymph Node; **NRRLN:** No Residual of Cervical Lymph Node; **ORR:** Objective Response Rate; **CR:** Complete Response;

**PR:** Partial Response; **SD:** Stable Disease.

**Table 3:** Protein expression correlation between Caspase-3, PARP and αV integrin in NPC tissue samples.

Factor	Expression	Total	Number of patients		*P* value
			αV integrin highexpression	αV integrin lowexpression	
			11	65	<0.001
	Low	29	19	10	
PARP	High	79	15	54	<0.001
	Low	26	20	6	

### αV Integrin is Differently Expressed in MCSs and MCs

**Figure 2:**
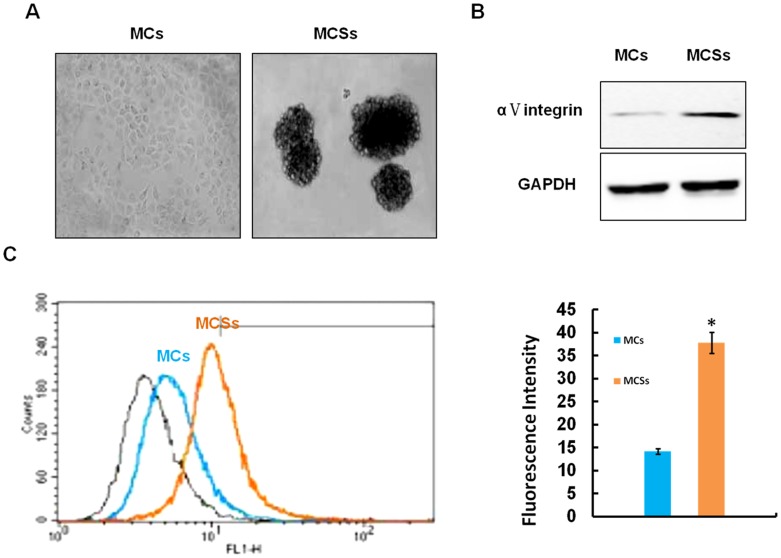
αV integrin levels in MCs and MCSs. **A:** Morphology of MCs and MCSs. **B:** The expressions of αV integrin in MCs and MCSs. **C:** The expressions of αV integrin in MCs and MCSs detected by Flow Cytometry. Values are means ± SEM, *P<0.01.

It has been demonstrated in our previous study that αV integrin is a critical factor mediating MCR to chemotherapy in MCSs [12]. We therefore hypothesized that the expression of αV integrin in NPC MCSs and MCs may be different. MCSs are cultured as previously described [12] (Fig. 2 A). As detected by flow cytometry assay and Western blot. The expression of αV integrin is much higher in MCSs than that in MCs (Fig. 2 B, C).

### Blocking the Function of αV Integrin Reversed Radioresistance of MCSs

**Figure 3:**
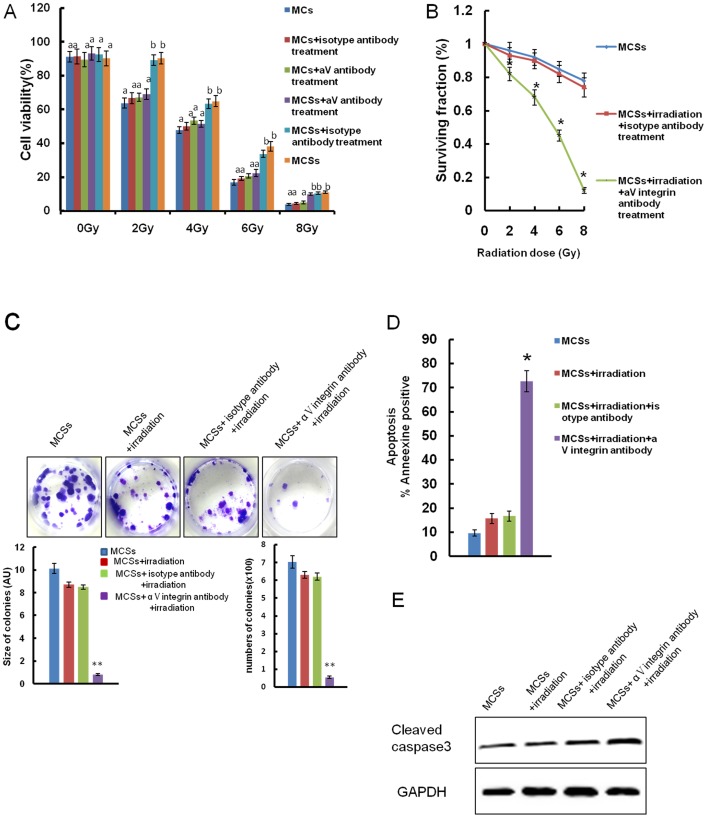
The effect of αV integrin on radiosensitivity of NPC cells in vitro. A: Cell survival of MCs and MCSs in presence of different dose of irradiation (0–8 Gy) combined with or without αV integrin blockade. Data are compared by one-way ANOVA Statistical, *p<0.01. **B:** Colony survival curves of MCSs in the presence of different dose of radiation (0–8 Gy) combined with or without αV integrin treatment respectively, *p<0.01. **C: **Images and quantification of the number and size of colonies formed from MCSs treated with or without αV integrin treatment in the presence of 2 Gy irradiation, *p<0.01. **D:** The Annexin-V assay of apoptosis for MCSs treated with or without αV integrin treatment in presence of irradiation, *p<0.01. Data are expressed by the percentage of cells that were stained with Annexin. E**:** The protein levels of cleaved caspase 3 in MCSs treated with or without αV integrin treatment in the presence of irradiation.

To determine if αV integrin is critical in multi-cellular radioresistance, we compared the cell survival rate of different groups with or without αV integrin function blockade in the presence of irradiation. It showed that blocking the function of αV integrin dramatically increased the radiosensitivity of MCSs (Fig. 3A), and more intriguingly, no changes of radiosensitivity were detected in MCs even after αV integrin blockade (Fig. 3A). Clonogenic survival assay was also performed to measure the radiation response. As shown in Figure 3 B, in the presence of irradiation, blocking the function of αV integrin in MCSs resulted in a significantly increased radiosensitivity relative to the control groups, indicating that αV integrin critically contribute to the radioresistance of MCSs. Meanwhile, αV integrin blocked MCSs resulted in a substantially decreased cell survival (Fig. 3C) and increased apoptosis (Fig. 3D) when exposed to 2 Gy fractionated irradiation. Additionally, the expressions of apoptotic genes cleaved Caspase-3 and cleaved PARP were found to be increased significantly in αV integrin blocked MCSs (Fig. 3E).

### SAPK/JNK Pathway is Involved in αV Integrin Mediated Multi-cellular Radioresistance of NPC MCSs

**Figure 4:**
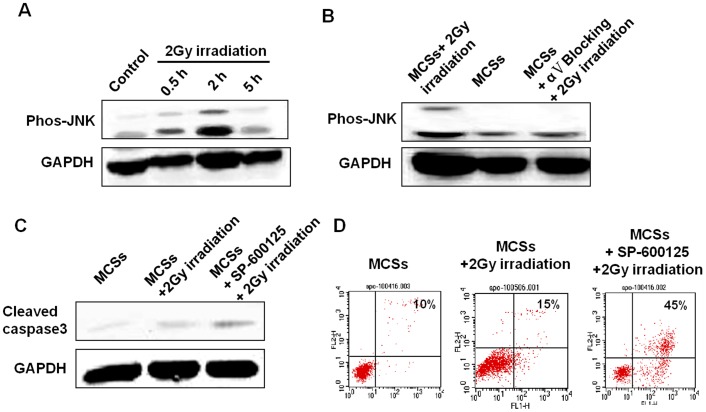
Molecular response of αV integrin mediate radioresistance in MCSs. **A:** Time-course level of phosphorylated JNK in presence of irradiation. **B:** The level of phosphorylated JNK 2 hrs after irradiation. **C:** The level of cleaved caspase 3 in MCSs treated with or without SAPK/JNK pathway inhibitor SP-600125. **D:** Apoptosis in MCSs treated with or without SAPK/JNK pathway inhibitor SP-600125.

Irradiation is a stress inducing apoptosis in cancer cells, and it is well known that SAPK/JNK pathway is a critical signaling activated by stress. To determine the mechanism mediating αV integrin’s inhibitory function on apoptosis, we investigated the effect of αV integrin on SAPK/JNK signaling pathways in MCSs. Western blotting showed that SAPK/JNK was substantially phosphorylated in MCSs of CNE-2 cells in response to irradiation (Fig. 4A). Blocking the function of αV integrin in MCSs substantially decreased the expression of phosphorylated JNK (Fig. 4B), and blocking of SAPK/JNK pathway increased the expression of cleaved casepase3 (Fig. 4C). Flow cytometry assay also showed that irradiation induced apoptosis of MCSs was increased by blocking SAPK/JNK pathway (Fig. 4D).

### αV Integrin Blocking Enhances the Radiosensitivity of NPC Xenografts

**Figure 5:**
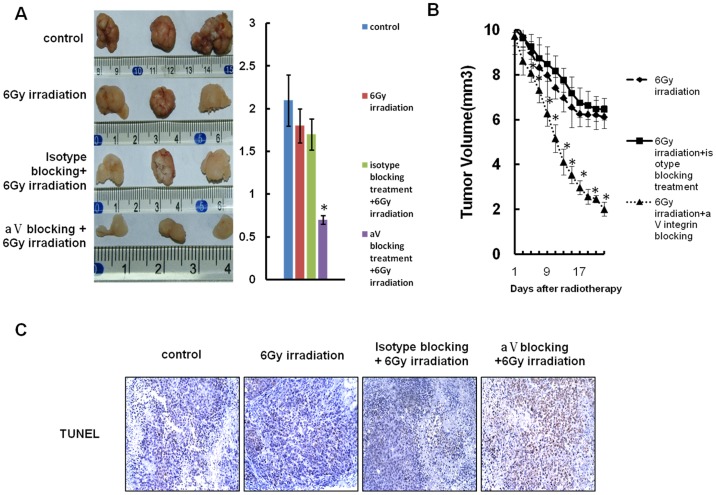
Effect of αV integrin blocking on radioresponse of NPC xenografts in mice with irradiation treatment. Same volume of MCSs (100 µl per mouse) were injected subcutaneously, Mice were treated with local radiation at a fractionated dose of 6 Gy combined with αV integrin blocking peptide or isotype blocking peptide peritumoral treatment (10 mg/Kg, twice a week) when the xenografts reached a mean size of 0.8–1 cm. Tumor weight was measured when the mice were sacrificed after 3 weeks. Each data point represents the mean weight of 3 tumors; **A:** Xenograft weight was measured when the mice were sacrificed 3 weeks after radiotherapy. A photograph of a representative xenograft in each group is also shown, *P<0.01. **B:** The decrease of xenograft size was measured every 2 days after radiotherapy, *p<0.01. **C:** Apoptosis detected by TUNEL assay was shown to compare the effect of αV integrin blocking peptide on radiosensitivity of in irradiated xenografts (×100).

To further confirm the effect of αV integrin on radiosensitivity of NPCs, we injected equal number (1.0×10^6^ per mouse) of CNE-2 cells subcutaneously into nude mice (3 mice for each group). The mice were exposed to 6 Gy fractionated irradiation, and a peritumoral injection of αV integrin blocking peptide or isotype blocking peptide (10 mg/kg, twice a week) were also administrated when the xenografts reached a mean diameter of 0.8–1.0 cm. The xenografts were excised and weighed 3 weeks after treatment. As shown in Fig. 5A and Fig. 5B, αV integrin blockade synergistically increased the effect of irradiation on xenografts. Xenografts were then fixed with 2% paraformaldehyde and dissected into sections at 8 µm. Immunochemistry staining of TUNEL was performed and found that the apoptosis of tumor in αV integrin blockade combined group is significantly higher than that in control groups (Fig. 5C). All of these indicate that αV integrin blockade may increase radiosensitivity of NPCs.

## Discussion

Previously, our group have found that downregulation of αV integrin promoted drug sensitivity in colorectal carcinoma multi-cellular spheroids [12]. We therefore propose that loss of αV integrin function also enhances multi-cellular radiosensitivity. Our present study shows that αV integrin also contributes to multi-cellular radioresistance in NPCs by exacerbating irradiation induced apoptosis. More significantly, the expressions of αV integrin in human NPC tumors negatively correlate to the levels of apoptosis related genes, highlighting the potential role of αV integrin-mediated anti-apoptosis reprogramming in human NPCs. Taken together, our data provide a mechanism whereby αV integrin acting as a tumor protector by regulating multi-cellular radioresistance in NPCs.

Our findings are consistent with the previous work showing that anti- aV integrin can enhance the efficacy of radiation therapy and reduce metastasis of human cancer xenografts in nude mice [13], [14]. More importantly and intriguingly, in our study, we present data to demonstrate that blocking the function of αV integrin in monolayers has little effect on their response to irradiation, indicating that αV integrin is only crucial for multi-cellular spheroids or biomass tumor in vivo. Furthermore, our studies have shed light on the mechanism through which αV integrin regulating apoptosis. Factors activating αV integrin are extensive, including intra- and extra-cellular factors, such as cytoskeleton, fibronectin, virus, force, shear stress, cell–cell adhesion, and cell-ECM adhesion [15]–[19]. In MCSs, cells adhere with each other and cell–cell junctions exist generally, leading to the hypothesis that αV integrin may be activated by cell–cell adhesion in MCSs and biomass tumor [20], [21]. Otherwise, cell adhesion might provide a precondition for facilitators to activate αV integrin. αV integrin has been thought of as a cell adhesion receptor regulating signal transduction pathways of cell proliferation, survival and apoptosis [22], [23]. Given cell proliferation, survival, and apoptosis are three of the most critical factors impacting radiosensitivity. [24]–[26]. This may be in part of the mechanism of activation of αV integrin in MCR.

Apoptosis is an unarguably common pathway to cell death initiating from irradiation [27], and NF-κB and JNK2 are two of the most important apoptotic factors, especially underlying stress [28]. It has already been demonstrated that αV integrin can activate NF-κB and inactivate JNK in some kinds of cells [29]. As a result in our study, we found that blocking SAPK/JNK pathway reversed radioresistance in MCSs, indicating that SAPK/JNK pathway is critical mediating MCR. It has been reported that SAPK/JNK pathway can be dramatically activated by endoplasmic reticulum stress (ER stress) [30], [31] and endoplasmic reticulum is well known to be the compartment of protein synthesis, including apoptotic related proteins. This correlation may explain how αV integrin blocking results in an increased expression of caspase 3 and PARP. Although we can not draw a conclusion that SAPK/JNK pathway is the only pathway triggered by αV integrin mediated multicellular radioresisitance, the evidence we got has given us a hint that SAPK/JNK pathway can be directly or indirectly activated by αV integrin. Our studies have revealed the profound impact of αV integrin on MCR to radiosensitivity, and it will be important for future work to examine the effect of αV integrin on each stage of NPC tumorigenesis in mechanistic detail.

The combination of molecular-targeted agents with irradiation is a highly promising avenue for cancer research and patient care. Given the role of αV integrin in mediating NPC radioresistance, αV integrin should be a potential target to improve the efficiency of radiosensitivity in NPCs.

## Materials and Methods

### Samples Collection

A tissue chip consisting of 105 human nasopharyngeal carcinoma (NPC) specimens was purchased from Shanghai Outdo Biotech Co.,Itd. A separated set of tissue specimens used for immunohistochemistry and Western blotting studies were collected from NPC patients who had undergone biopsies at Southwest Hospital under a protocol approved by Southwest Hospital. The **Objective Response Rate** (ORR) and histological subtypes were defined by an oncologist (pathologist) in the Southwest Cancer Center, Southwest Hospital. **Complete Response** (**CR**) means all detectable tumor has disappeared; **Partial Response** (**PR**) corresponds to at least a 50% decrease in the total tumor volume but with evidence of some residual disease still remaining; **Stable Disease** (**SD**) means the tumors stay the same size, to account for measurement errors on scans and to discount “insignificant” changes, stable disease includes either a small amount of growth (typically less than 20 or 25%) or a small amount of shrinkage (anything less than a PR unless minor responses are broken out). Radiosensitive patients are clarified as those reached CR 2 to 4 weeks after irradiation therapy (GTV>70 Gy), and radioresistant patients are clarified as those of PR or SD or even with disease progression 2 to 4 weeks after irradiation therapy (GTV>70 Gy).

### Assessment of Immunohistochemical Staining

Immunohistochemical staining was scored as 0–4. No staining or weak staining were scored was 0 and 1, respectively. Strong staining of 25% tumor cells or moderate staining of <80% scored 2. Strong staining of 25–50% or moderate staining of >80%, and strong staining of >50% tumor cells, scored 3 and 4, respectively. Ten representative areas were counted in each case from high power fields. Slides were examined and scored independently by 2 researchers blinded to other pathological information.

### Cell Culture

CNE-2 cells were routinely grown and passaged as monolayers in RPMI1640 medium (HyClone, UK) supplemented with 5% fetal bovine serum, penicillin (100 IU/mL), and streptomycin (100 µg/mL) under a humidified atmosphere of 5% CO_2_ at 37°C. MCSs were obtained by using the liquid overlay technique. Exponentially-growing CNE-2 cells were added in culture medium in plates which were previously coated with 2% agarose. The plates were gently horizontally swirled 10 min every 3 h in the first 24 h, then 10 min every 4 h. Appropriate medium was refreshed every other day. For antibody treatment, cells were incubated with purified endotoxin-free mAbs (175 µg/ml) for 24 h.

### Western Blotting

Cells were washed with phosphate-buffered saline (PBS) and lysed at 4°C. in 2×SDS loading buffer (0.1 M Tris–HCl [pH 6.8], 0.2 M DTT, 4% SDS, 20% glycerol, and 0.2% bromophenol blue). Protein was quantitated by using the RC DC protein assay (BIO-RAD, CA, USA), resolved by 8% SDS–PAGE, and transferred to nitrocellulose membranes (Qbiogene, UK). Target protein was detected by anti-αV integrin (Santa Cruz Biotechnology, Santa Cruz CA, USA), anti-SAPK/JNK antibody (Cell Signaling Technology), anti-phospho-SAPK/JNK antibody (Cell Signaling Technology), anti-cleaved caspase-3 (Santa cruz), goat polyclonal antibody against cleaved caspase-9 (Santa cruz) and rabbit polyclonal antibody against cleaved poly ADP-ribose polymerase (PARP) (Santa cruz). After washing and incubating with secondary antibodies, immunoreactive proteins were visualized by the Enhanced Chemiluminescnet Substrate (Pierce, IL, USA).

### Cell Survival Assay

Cell survival was evaluated by using the cell counting kit 8 (Dojindo Laboratories, Japan). In contrast to monolayers, MCSs were digested by Non-enzyme Cell Detach Solution (Applygen Technologies, China) for 10 min before using the cell counting kit 8 to detect cell survival.

### Colony Survival Assay

Cells were seeded into 24-well culture dishes in triplicates (1000 cells to each well). The cells were allowed to form colonies during 1 week, and then cells were treated with different doses of 6MV X-ray radiation [IBL 637; Cis-Bio International, Gif-sur-Yvette, France]. The radiation doses were 0, 2, 4, 6 and 8 Gy, respectively; the dose efficiency was 300 cGy/min. After an incubation period of 12–15 days, the colonies were fixed with methanol and stained with crystal violet. Colonies of >50 cells were counted and analyzed.

### Flow Cytometry Analysis of Apoptosis

Flow cytometry was performed to detect apoptosis of trypsin- dissociated cells with AnnixinV-PE apoptosis Detection Kit (Beyotime). Cells were washed and resuspended in 0.5 ml PBS buffer, and fixed for 24 hr in 70% alcohol. Annixin V- PE (50 µg/ml) was added and incubated for 30 min on ice, and then analyzed by FCM (FACScan, Becton Dickinson, San Jose, CA).

### In vivo Study

Female BALB/c (nu/nu) nude mice, 4–5 weeks old, weighing 17–22 g, were housed in filter-capped cages kept in a sterile facility and maintained in a specific pathogen-free barrier system. After 3 weeks, xenografts established by subcutaneous injection CNE-2 MCSs in mouse hips reached a mean diameter of 0.8–1.0 cm, and then 6 Gy fractionated irradiation combined with or without daily peritumoral injection of αV integrin blocking peptide or isotype blocking peptide (Santa Cruz Biotechnology, Santa Cruz CA, USA) were administrated (10 mg/Kg, twice a week). Mice were sacrificed 3 weeks later and the xenografts were excised and weighed.

### Statistical Analysis

Statistical analysis was performed with SPSS (SPSS, Chicago, IL) using Students t-test or one-way ANOVA. Differences were considered statistically significant when P-values were less than 0.05. Error bars represent standard error of the mean.
